# Assessment of cardiac adverse events following COVID-19 vaccination by speckle tracking echocardiography

**DOI:** 10.1038/s41598-024-61641-y

**Published:** 2024-05-13

**Authors:** Srisakul Chaichuum, Ching-Li Tseng, Su-Chen Chang, Chih-Lin Chan, Chu-Ying Hsu, Edward Chiang, Masao Daimon, Shuo-Ju Chiang, Hsiang-Ho Chen

**Affiliations:** 1 Graduate Institute of Biomedical Materials and Tissue Engineering, Taipei Medical University, Taipei, Taiwan; 2 Division of Cardiology, Department of Internal Medicine, Taipei City Hospital Yangming Branch, Taipei, Taiwan; 3 School of Medicine, Chung Shan Medical University, Taichung, Taiwan; 4 Department of Cardiovascular Medicine, The University of Tokyo Hospital, Tokyo, Japan; 5 Graduate Institute of Biomedical Engineering, Center for Biomedical Engineering, College of Engineering, Chang Gung University, Taoyuan, Taiwan; 6 Department of Plastic and Reconstructive Surgery, Chang Gung Memorial Hospital, Taoyuan, Taiwan

**Keywords:** COVID-19 vaccine, Cardiac-related adverse event, Subclinical myocardial dysfunction, Tissue speckle tracking, Myocardial strains, Medical imaging, Predictive markers

## Abstract

Cardiac discomfort has been reported periodically in COVID-19-vaccinated individuals. Thus, this study aimed to evaluate the role of myocardial strains in the early assessment of the clinical presentations after COVID-19 vaccination. Totally, 121 subjects who received at least one dose of vaccine within 6 weeks underwent laboratory tests, electrocardiogram (ECG), and echocardiogram. Two-dimensional speckle tracking echocardiography (2D-STE) was implemented to analyze changes in the left ventricular myocardium. After vaccination, 66 individuals (55.4 ± 17.4 years) developed cardiac discomforts, such as chest tightness, palpitations, dyspnea, and chest pain. The ECG readings exhibited both premature ventricular contractions and premature atrial contractions (n = 24, 36.4%), while none of the individuals in the control group manifested signs of cardiac arrhythmia. All had normal serum levels of creatine phosphokinase, creatine kinase myocardial band, troponin, N-terminal pro b-type natriuretic peptide, platelets, and D-dimer. Left ventricular ejection fraction in the symptomatic group (71.41% ± 7.12%) and the control group (72.18% ± 5.11%) (p = 0.492) were normal. Use of 2D-STE presented global longitudinal strain (GLS) and global circumferential strain (GCS) was reduced in the symptomatic group (17.86% ± 3.22% and 18.37% ± 5.22%) compared to the control group (19.54% ± 2.18% and 20.73% ± 4.09%) (p = 0.001 and p = 0.028). COVID-19 vaccine-related cardiac adverse effects can be assessed early by 2D-STE. The prognostic implications of GLS and GCS enable the evaluation of subtle changes in myocardial function after vaccination.

## Introduction

The World Health Organization reported that coronavirus disease 2019 (COVID-19) reached pandemic levels in March 2020. There have been over 660 million globally documented and confirmed cases, including approximately 6.69 million people who died as of January 11, 2023. The severe acute respiratory syndrome coronavirus 2 (SARS-CoV-2) that causes COVID-19 has caused repeated waves of outbreaks across the globe. According to viral characteristics, tissue tropism of the virus varies depending on the permissiveness and susceptibility of the host cell. The primary site of SARS-CoV-2 tropism is the lung^1^. The SARS‐CoV‐2 spike (S) protein, a transmembrane fusion protein, interacts with the human angiotensin‐converting enzyme 2 (ACE2) receptor for host cell entry^2^. The formation of ACE2 takes place through pathways mediated by various proteases including cathepsin G, chymase, kallikrein, neutral endopeptidase, tonin, and tryptensin. These proteases are localized in specific organs and tissues, such as the lungs, arterioles, kidney, brain, and myocardium^3,4^. Regulation of the renin-angiotensin system (RAS), which has ACE2 as a mediator, influences coagulation, some inflammatory mechanisms, and control of blood pressure. An imbalance in ACE2 could potentially increase vasoconstriction, blood pressure, and pro-inflammatory molecules^5^. The clinical manifestations of COVID-19 infection occur primarily in the upper respiratory tract, causing severe ‘flu’-like symptoms. Disease progression can lead to acute respiratory distress, renal failure, and long-term ramifications, such as myocarditis, and death^6,7^.

Vaccines provide a significant degree of reduction in the risk of infection and protection against serious illness. The development of vaccines was accelerated to serve as a promising strategy to prevent death from this pandemic. The urgent need for COVID-19 vaccines shortened the research and development processes and drove it to be marketed with emergency use authorization. Various technologies were applied to develop different vaccine types including component viral vaccines and whole viral vaccines. The platforms of protein subunit, virus-like particle, DNA-based, RNA-based, non-replicating viral vector, and replicating viral vector were designed to use SAR-CoV-2 component viral vaccines, whereas inactivated vaccine and live attenuated vaccine used the copy of the whole virus. Nucleic acid vaccine was accounted for the stage-of-the-art development fighting against COVID-19 infection^8,9^. It was recently reported that a total of 9.4 billion vaccine doses have been administered, and 50.3% of the population worldwide is fully vaccinated^10^. However, several adverse events (AEs) have been reported after COVID-19 vaccine administration. Myocarditis, which is inflammation of the myocardium, has been noted as a sporadic side effect of the vaccine. The United States Centers for Disease Control and Prevention (CDC) noted that there were 11 verified reports of myocarditis among males between the ages of 12 and 29 years^11^. Salah and Mehta described 8 reports retrieved in a PubMed/Medline search on ‘COVID-19 vaccine’ and ‘myocarditis’ through June 27, 2021. Among the 15 total patients included in those 8 studies, myocarditis was associated with the Pfizer-BioNTech messenger RNA (mRNA) vaccine in 60% of the cases and with the Moderna mRNA vaccine in 33% of the cases^12^. The United States Food and Drug Administration’s Vaccine Adverse Event Reporting System (VAERS) database was reviewed for possible immune-related adverse events (irAEs) following vaccination. Among 81 patients with cancer who received immune checkpoint inhibitors (ICIs), 6 developed clinical presentations suspicious for irAEs, including myositis and cardiogenic shock, after administration of a COVID-19 mRNA vaccine^13^.

In our clinical practice, we found that several patients who received a COVID-19 vaccine developed cardiac-related discomfort that did not meet the diagnostic criteria of myocarditis. Angeli et al. described the occurrence of an adverse reaction, hypertension after COVID-19 vaccination. According to the mechanism of the mRNA vaccine, free-floating S protein might expand a massive interaction with the ACE2 receptors leading to receptor degradation, which would subsequently contribute to hypertension^14^. Hence, a relationship between cardiac discomfort and COVID-19 vaccination is probable. The tissue tracking technique is a novel extension of the Doppler method that focuses on cardiac function, which allows identification of major cardiac changes in symptomatic individuals. Two-dimensional speckle tracking echocardiography (2D-STE) has been regarded as a promising tool for the evaluation of heart failure, coronary artery disease, myocardial dyssynchrony, valvular heart disease, and arrhythmia^15–19^. This modality is easily accessible, cost-effective, non-radiative, and non-invasive, with high sensitivity and specificity for LV functional assessment, and it can detect subclinical myocardial dysfunction early in the disease process^20–22^. In this regard, we hypothesized that the global strain of the myocardium, a cardiac mechanical parameter, can be used to assess subtle structural and functional changes indicating cardiac AEs in patients who received COVID-19 vaccines. This study aimed to evaluate the role of cardiac mechanics, particularly myocardial strain in the early assessment of the clinical presentations of cardiac manifestations after COVID-19 vaccine administration.

## Materials and methods

### Study population

The cross-sectional study was conducted in the Taipei City Hospital Yangming Branch from September 2021 until February 2022. According to the Ministry of Public Health Policy of COVID-19 pandemic management in Taiwan, the population was encouraged to receive the first dose of the vaccine and the consecutive dose as prescribed period. Therefore, the population voluntarily received vaccination against the coronavirus. The record of COVID-19 vaccination of the population was documented by Taiwan National Health Insurance Administration. The inclusion criteria for symptomatic and control groups were subjects aged > 20 years and < 75 years with no history of COVID-19 infection. In total, 121 subjects who received COVID-19 vaccines at least one dosage within 6 weeks, were selected. Individuals who visited the outpatient cardiovascular clinic due to cardiac discomfort such as chest tightness, chest pain, palpitations, and dyspnea after vaccination, were recruited and classified as a symptomatic group. Structured clinical interviews were conducted by physicians, including questions regarding physiological symptoms, frequency, duration, severity, range, and patient description, modified from the patient-reported outcome measure published by Moshkovich et al.^23^. The patients underwent laboratory testing, physical examination, 12-lead electrocardiogram (ECG), and transthoracic echocardiography (TTE). Separately, individuals who came to the general health checkup center and underwent ECG and echocardiogram as the routine checkup program, were recruited and classified as a control group. Controls had no history of cardiovascular chronic diseases and had noncardiac discomfort after vaccination. Numbers and dates of vaccinations were collected for each individual. The symptoms following COVID-19 vaccination, such as chest tightness, chest pain, dyspnea, palpitations, and foot edema were assessed. Chronic diseases including diabetes, hypercholesterolemia, hypertension, and chronic kidney disease were recorded during the clinical interview. Current medications and cardiovascular risk factors, for instance, smoking and family history of stroke, cardiac, and other major events, were also recorded. The exclusion criteria were patients with a history of acute (within 3 months) and chronic coronary syndrome, acute heart failure, documented coronary artery disease, regional wall motion abnormality, and left ventricular ejection fraction (LVEF) less than 50%. Cases with hemodialysis, significant valvular disease or a prosthetic valve, permanent pacemaker, unstable hemodynamic condition, terminal major organ disease, cancer in status, psychological disorder, and inadequate quality ultrasound images were also excluded. The study was approved by the Institutional Review Board of Taipei City Hospital (TCHIRB-11101001). The participants provided their written informed consent.

### Laboratory examination

All patients underwent serological examinations. Laboratory data included thrombosis factors (platelet count and D-dimer) and cardiac inflammatory markers, such as creatine phosphokinase (CPK), creatine kinase myocardial band (CK-MB), Troponin T (TnT) and N-terminal pro b-type natriuretic peptide (NT-proBNP). Incidents of cardiac-related discomfort after vaccination were collected. Data validation was evaluated by two independent investigators to ensure the accuracy and consistency of the analysis.

### Transthoracic echocardiography

Comprehensive TTE was performed by experienced sonographers, within 6 weeks following vaccine administration. Vivid E95 (GE Healthcare, Horten, Norway) equipped with M5S was used for evaluation. In all individuals, standard 2D images, the apical 4-, 3-, and 2-chamber views (A4C, A3C, and A2C, respectively), and the short-axis level of the mitral valve (SAX-MV) and level of papillary muscles (SAX-PM), consisting of three cardiac cycles actuated to the QRS complex, were captured and analyzed. Conventional 2-D parameters were collected and analyzed^24,25^. Left ventricular function was assessed according to the guideline^26^. Simpson’s biplane method was applied to calculate a LVEF. Preserved and normal LVEF were indicated as 50–60% and > 60%, respectively. Peak velocities of early (E) and late (A) diastolic flow, and the E/A ratio were measured using pulsed-wave interrogation of the mitral valve inflow. The tissue Doppler technique was performed to evaluate septal mitral annular motion. Early septal diastolic annular velocity (e′) based on tissue Doppler imaging was measured. An indicator of a primary cardiac event, the E/e′ ratio was calculated^27^.

### Tissue speckle tracking

The standard echocardiographic views on three consecutive beats in A4C, A3C, and A2C were used for longitudinal strain analysis. In addition, SAX-MV and SAX-PM were used for circumferential strain analysis of the LV using 2D speckle tracking software (EchoPAC, GE Healthcare, Horten, Norway). Cardiac strain is a measure of the deformation that occurs when two neighboring points on the myocardium move at different velocities, resulting in a change in myocardial shape. Lengthening (positive strains) and shortening (negative strains) of the myocardium contribute to the strain values presented as percentages (%). The feature of myocardial tracking was achieved when adequate TTE examinations, defined as good image quality and an optimum frame rate of 50–70 frames per second, were obtained. The recorded images were analyzed offline. A region of interest was defined at end-diastole by manual outline. The myocardium was divided into six segments in each view. Regions of the border were individually adjusted throughout so that the whole myocardium was correctly tracked. The wall thickness was also manually adjusted if necessary for complete analysis^28^. Myocardial strains were applied to evaluate myocardial deformation in the longitudinal plane, which is displayed in an 18-segment LV model, and in the circumferential plane, which is displayed in a 12-segment LV model. The regional wall segments in a series of longitudinal views were composed of basal septal, mid septal, apical septal, apical lateral, and basal lateral segments of the LV. The regional wall segments in a series of circumferential views were derived from the SAX-MV view, which is composed of the basal anterior, basal anteroseptal, basal inferoseptal, basal inferior, basal inferolateral, and basal anterolateral segments and from the SAX-PM view, which is composed of the mid anterior, mid anteroseptal, mid inferoseptal, mid inferior, mid inferolateral, and mid anterolateral segments of the LV^29^. During systole and diastole, the myocardium shortens and lengthens the wall muscles in the longitudinal and circumferential planes. Inter-observer reproducibility was examined by random selection of 20 strain analysis studies (10 GLS, 10 GCS). The second investigator independently repeated the analysis of global strains after a month to minimize measurement bias. To assess assuming a random effect model, intraobserver Standard Error Measurement (SEM_intra_) was applied to express the random error by a regular observer. Intraobserver Minimal Detectable Change (MDC_intra_) was performed to evaluate the smallest change beyond random measurement error at a level of confidence of 95%^30^.

### Statistical analysis

All categorical variables are presented as proportions or percentages. For data that were distributed normally, continuous variables are presented as mean ± standard deviation. For data that were not normally distributed, a median with an interquartile range was used. Comparisons of categorical variables between the control and symptomatic groups were investigated using the X^2^ test. Continuous variables were compared using an independent t-test, as indicated. SEM was quantified by SD × √(1 − ICC); ICC, Intraclass Correlation Coefficient. The formula to calculate MDC was 1.96 × √2 × SEM. A p-value < 0.05 was considered statistically significant. The statistical analysis was performed using SPSS software, version 21.0 (IBM Corporation, NY, USA).

### Institutional review board statement

The study was conducted following the Declaration of Helsinki and approved by The Institutional Review Board of Taipei City Hospital (TCHIRB-1020802-E).

### Informed consent

Informed consent was obtained from all subjects involved in the study. Written informed consent has been obtained from the patients to publish this paper.

## Results

### Patient characteristics and cardiac biomarkers

In total, 121 patients who met the inclusion criteria and for whom adequate image quality for tissue tracking analysis was obtained were recruited for this study. Patients who were vaccinated and developed cardiac-related discomfort according to verbal inquiry and medical history questionnaire and clinical interview (n = 66; mean age, 55.4 ± 17.4 years) were classified as a symptomatic group and those who were recruited from health check center and vaccinated without developing adverse cardiac outcomes (n = 55; mean age, 53.7 ± 14.8 years) were classified as a control group. There were no differences between study groups in sex and ethnicity. Among patients in the cardiac AEs group, clinical symptoms reported included chest tightness in 89% (n = 59), palpitations in 35% (n = 23), dyspnea in 32% (n = 21), and chest pain in 9% (n = 6). In ECG readings, premature atrial contractions (PACs) and premature ventricular contractions (PVCs) (total n = 24, 36.4%) and sinus tachycardia (n = 20, 30.3%) were detected in these patients, while none of the individuals in the control group manifested signs of cardiac arrhythmia or sinus tachycardia. Nonetheless, there were no significant differences in baseline clinical characteristics and current medications between the symptomatic and control groups. In total, 190 doses of vaccines against COVID-19 infection were used. Each individual in the symptomatic and control groups received the first dosage (n = 66 and n = 55, respectively). There were 34 vs 27-s booster doses, and 6 vs 2-third booster shots of vaccines were recorded from symptomatic and control groups, respectively. For the vaccination profile including the number of vaccine doses administered, there was no difference between groups. For the list of vaccines used among symptomatic and control groups, AstraZeneca (n = 37, 34.6% vs n = 29, 34.9%), Moderna (n = 36, 33.6% vs n = 30, 36.1%), BioNTech (Pfizer) (n = 30, 28% vs n = 21, 25.5%), and Medigen (n = 4, 3.7% vs n = 3, 3.6%) vaccines were provided and recorded by the government. The period from the last vaccination of patients in symptomatic and control groups was not significantly different (27 ± 6 days vs 32 ± 5 days, respectively; p = 0.641), as shown in Table 1. The mean serological biomarker levels in patients with cardiac AEs, including platelets (222.0 × 10^3^/μL; interquartile range [IQR] 199.2–275.5), D-dimer (0.3 mg/L; IQR 0.1–0.4), CPK (93.0 U/L; IQR 66.5–137.5), CK-MB (10.4 U/L; IQR 2.3–14.7), TnT (6.0 ng/L; IQR 4.0–8.5), and NT-proBNP (44.5 pg/mL; IQR 20.2–79.5), were normal.

**Table 1:** Comparison of demographic data, echocardiographic examinations, and myocardial strain analysis in the group of patients with cardiac adverse events (AEs) and the control group.

Patient characteristics	Cardiac AEs (n = 66)	Control (n = 55)	*p*-value
Gender, male/female, n	27/39	21/34	0.760
Age, years	55.4 ± 17.4	53.7 ± 14.8	0.076
BMI, kg/m^2^	26.4 ± 4	24.5 ± 2.7	0.241
Diabetes, n (%)	5 (7)	3 (5)	0.583
Hypercholesterolemia, n (%)	11 (16)	5 (9)	0.298
Hypertension, n (%)	8 (12)	9 (13)	0.155
Vaccination profile			
Second vaccination, n (%)	34 (47)	27 (62)	0.729
Third vaccination, n (%)	6 (9)	2 (5.4)	0.318
Period from last vaccination, days	27 ± 6	32 ± 5	0.641
Medication			
ARB, n (%)	2 (3)	3 (5.4)	0.789
CCB, n (%)	4 (6)	4 (7.3)	0.878
Statin, n (%)	7 (10.6)	4 (7.3)	0.513
β-blocker, n (%)	1 (1.5)	2 (3.6)	0.924
DPP-4 inhibitor, n (%)	2 (3)	1 (1.8)	0.856
Metformin, n (%)	3 (4.5)	2 (3.6)	0.820
Echocardiographic examination			
IVSd, cm	0.94 ± 0.24	0.80 ± 0.22	**0.002**
LVIDd, cm	3.93 ± 0.58	3.87 ± 0.54	0.545
LVPWd, cm	1.15 ± 0.83	0.97 ± 0.19	0.144
LVIDs, cm	2.56 ± 0.42	2.52 ± 0.36	0.646
LVEF, %	71.41 ± 7.12	72.18 ± 5.11	0.492
E vel, m/s	0.73 ± 0.17	0.78 ± 0.19	0.139
A vel, m/s	0.74 ± 0.19	0.69 ± 0.18	0.126
E/A ratio	1.08 ± 0.45	1.24 ± 0.56	0.096
eʹ, cm/s	8.64 ± 3.96	10.25 ± 3.58	**0.023**
E/e′	9.32 ± 3.56	7.74 ± 2.34	**0.005**
Myocardial strain analysis			
GLS, -%	17.86 ± 3.22	19.54 ± 2.18	**0.001**
GCS, -%	18.37 ± 5.22	20.73 ± 4.09	**0.028**

*BMI* body mass index, *ARB* aldosterone receptor blockers, *CCB* Calcium-channel blocker: DPP-4 inhibitor, dipeptidyl peptidase-4 inhibitor; *IVSd* interventricular septal width in diastole; *LVIDd* left ventricular internal dimension in diastole; *LVPWd* left ventricular posterior wall width in diastole; *LVIDs* left ventricular internal dimension in systole; *LVEF* left ventricular ejection fraction; *E vel* early mitral flow velocity; *A vel* late mitral flow velocity; *E/A* the ratio of mitral peak velocity of the early filling (E) to peak velocity of the late filling (A); *eʹ* early diastolic mitral annular velocity; *E/e′*, the ratio of mitral peak velocity of the early filling (E) to early diastolic mitral annular velocity (eʹ); *GLS* global longitudinal strain; and GCS, global circumferential strain.

*Data are presented as mean ± SD or number (percentage).

### Echocardiographic measurement

Baseline echocardiographic data are presented in Table 1. In total, none of the subjects had evidence of echocardiographic regional wall motion abnormalities. Patients in the symptomatic group had thicker interventricular septal width in diastole (IVSd) than those in the control group (0.94 ± 0.24 cm vs 0.80 ± 0.22 cm, respectively; p = 0.002). Moreover, patients in the symptomatic group had a greater ratio of mitral peak velocity of the early filling (E) to early diastolic mitral annular velocity (eʹ) (E/e′) than those in the normal group (9.32 ± 3.56 vs 7.74 ± 2.34, respectively; p = 0.005), which was in accord with e’ (8.64 ± 3.96 vs 10.25 ± 3.58, respectively; p = 0.023). Despite that, most patients had normal LVEF at a visit, in both the cardiac AEs (71.41% ± 7.12%) and the control group (72.18% ± 5.11%) (p = 0.492). There were no significant differences in other echocardiographic parameters between subjects who developed cardiac-related discomfort and those who did not show any cardiovascular side effects after COVID-19 vaccination.

### Myocardial strain

**Figure 1:**
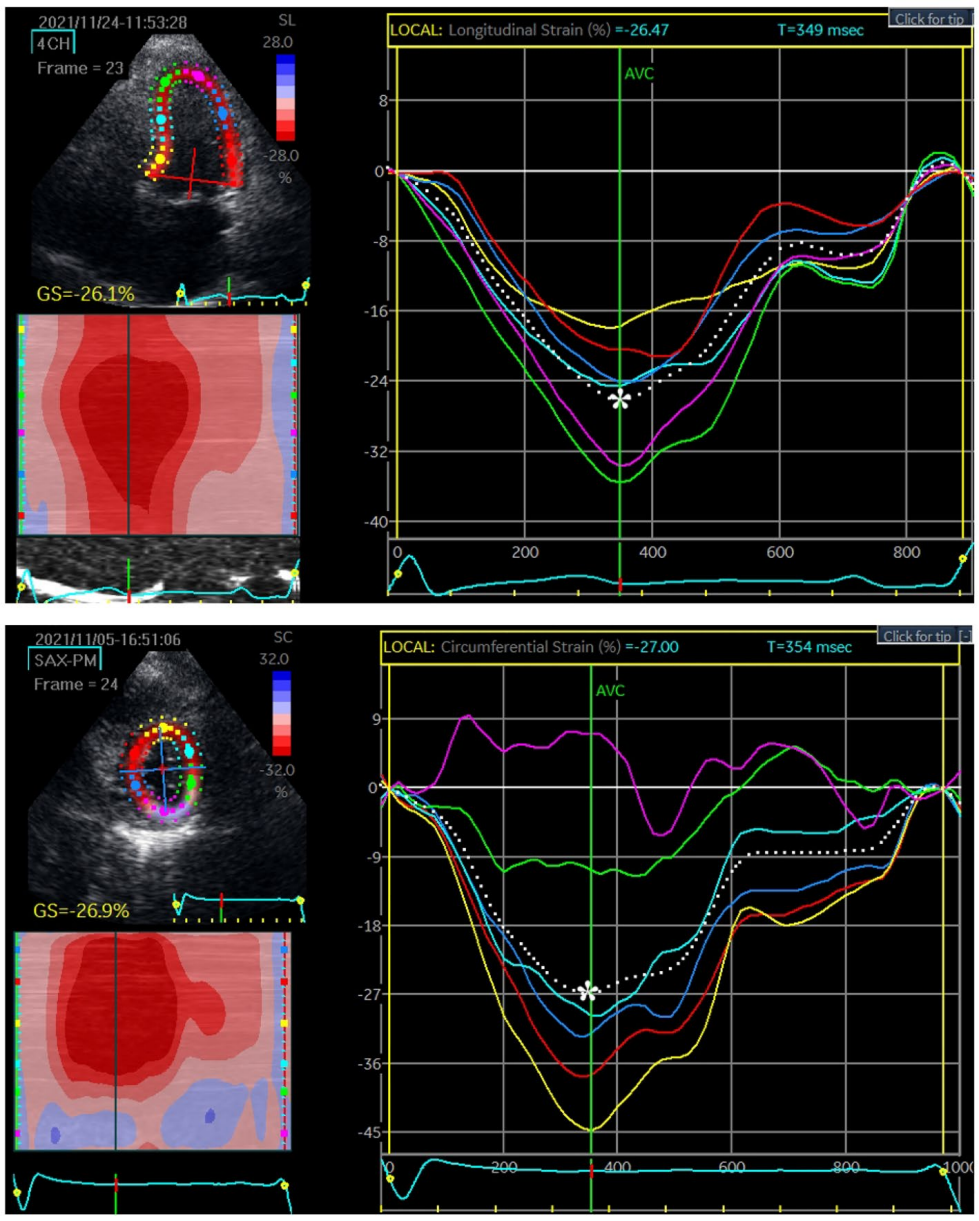
Representative global longitudinal strain (GLS) and global circumferential strain (GCS) analysis. The myocardial strains were quantified as the peak systolic value in each myocardial segment. The upper and lower figures represent the peak systolic GLS and GCS, respectively. The most negative values, which are denoted by the white asterisks, were measured prior to aortic valve closure (AVC).

**Figure 2:**
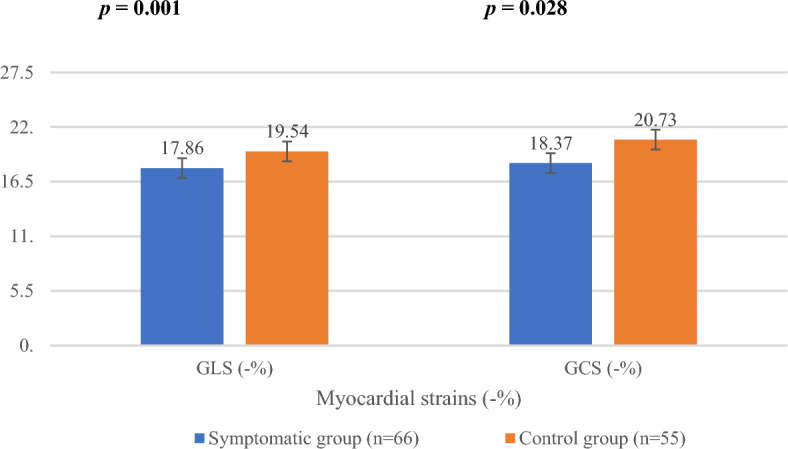
Comparison of global longitudinal strain (GLS) and global circumferential strain (GCS) between symptomatic and control groups. Strains are presented as a percentage.

EchoPAC software was implemented to analyze myocardial strain. Representative cases of longitudinal strain and circumferential strain with quantification are presented in Fig. 1. There were 55 vaccinated patients in the control group, and 66 vaccinated patients with cardiac AEs were evaluated for myocardial deformation to quantify GLS and GCS. The GLS in the case group (17.86% ± 3.22%) was significantly reduced compared with that in the control group (19.54% ± 2.18%) (p = 0.001). Similarly, the GCS in patients who developed cardiac discomfort (18.37% ± 5.22%) was also significantly lower than that in patients in the control group (20.73% ± 4.09%) (p = 0.028). The results demonstrated that GLS and GCS could reveal subclinical dysfunction of the myocardium (Fig. 2). On repeat reads of interobserver reproducibility for the measurements of GLS and GCS were 97.2% and 96.5%, respectively. The SEM_intra_ was 1.89 strain points (1.89%) for GLS and 2.26 strain points (2.26%) for GCS. This yielded an MDC_intra_ of 5.23% for GLS and 6.26% for GCS.

## Discussion

Our study was conducted in Taiwan since the early period of the COVID-19 pandemic. There were only a small number of COVID-19 infected patients here. None of our study population had the previous infection. Our study results showed that global myocardial strain, including longitudinal strain and circumferential strain, are sensitive, non-invasive, and reproducible indices that are clinically relevant to the subtle changes in the left ventricular myocardium that may occur as a consequence of the cardiac impact of COVID-19 vaccination. Hence, we found that the potential capability of tissue speckle tracking analysis can evaluate and indicate early myocardial dysfunction and adverse cardiac events after immunization with COVID-19 vaccines.

Various vaccine designs have been developed to elicit an immune response, which can be classified by the host-cell translation process. Non-replicating viral vector vaccines, mRNA vaccines, and protein subunit vaccines undergo the translation of nucleic acid into a modified S protein within the host cell to further enhance both humoral and cellular immunity^31^. Harrison et al. mentioned that the ACE2 receptor has a role as a cardiac function regulator, and is generally expressed in coronary endothelial cells, cardiac fibroblasts, and cardiomyocytes^32^. According to the mechanism of host cell entry in natural SARS-CoV-2 infection, it is possible that S proteins expressed by a vaccine can bind to ACE2 receptors and subsequently be internalized. Once clearance of the S protein with bound ACE2 by antibodies and immune complexes begins, RAS imbalance and enzymatic activity could reduce the overall availability of ACE2, eventually inducing vasoconstriction and increased blood pressure, leading to a burden on the heart, such as elevation of intraventricular volume and pressure, impacting ventricular function. Concurrently, this clearance elicits the formation of immune complexes, which would subsequently be eliminated by the macrophage-monocyte network and induce inflammation. This phenomenon could lead to serious consequences on the vessels and heart, particularly in vulnerable groups, such as patients with hypertension, arterial aneurysm, and atherosclerosis^5,33^.

In terms of disease pathogenesis, SARS‐CoV‐2 may cause a variety of cardiac complications, including acute myocardial infarction, acute heart failure, malignant arrhythmia, myocarditis, and finally, cardiogenic shock^34^. SARS‐CoV‐2 also causes endotheliopathy, which is related to systemic inflammation, cytokine storm, oxidative stress, and coagulopathy. This occurrence triggers an exaggerated immune response associated with microvascular dysfunction, tissue ischemia, and rapid deterioration^35^. A report from the beginning of the COVID-19 outbreak through June 30, 2020 mentioned that there were 14 identified cases with myocarditis/pericarditis occurring secondarily to the infection^36^. Moreover, a study from the SARS-CoV outbreak in Toronto, Canada in 2003 showed that viral RNA was observed in 35% of autopsied hearts^37^. A correlation of circulating ACE2 with susceptibility to SAR-CoV infection was revealed in a previous study. The progression of heart failure after viral infection can be accelerated by ACE2 receptor downregulation or soluble ACE2 accumulation. On the contrary, overexpression of ACE2 receptors can forestall or even reverse the heart failure phenotype. However, the evidence indicating decreased ACE2 expression resulting from SAR-CoV-2 infection in the heart and a direct relationship between viral infection and level of ACE2 expression remains unclear^38,39^. An underlying mechanism of SAR-CoV-2 infection involving RAS/angiotensin-converting enzyme (RAS/ACE) classical and alternative pathways, which also take place in the myocardium, was suggested in a previous study. The RAS has a significant role in the cardiovascular system, influencing blood pressure, heart remodeling, and heart function.

As stated in several anecdotal reports of cardiac manifestations after COVID-19 vaccination, potential interactions with the cardiovascular system have been considered. However, myocarditis was not observed in our population study. The majority of patients had normal cardiac inflammatory markers such as CPK, CK-MB, TnT, and NT-proBNP. To evaluate structural and functional changes resulting from cardiac dysfunction, TTE is primarily used^40,41^. The TTE examination in our study showed that the entire group of patients who developed cardiac discomfort after vaccine administration presented with a normal LVEF, a traditional indicator for heart pumping performance. Our finding is concordant with that of Claus et al., who explained that in circumstances when LVEF was in the normal range, longitudinal strain could represent fiber dysfunction at the level of the subendocardium, whereas circumferential strain is more specific to functional changes at the subepicardial fiber layer^42^. Furthermore, patients who had cardiac-related discomfort after vaccination had thicker IVSd than normal subjects. This implicates LV remodeling, which is related to basal septal hypertrophy (BSH), an early stage in advance of heart failure. Similarly, Yalçin et al. described that BSH was associated with the progression of stressed heart morphology^43^. Therefore, the global LV remodeling could reveal a consequence of COVID vaccination-induced RAS imbalance. The cardiac mechanical parameter, myocardial deformation, is reflected by GLS, an important tool evaluated by 2D-STE. Several studies demonstrated that GLS is one of the parameters hinting at cardiac injury. Significantly lower GLS corresponded to a risk of adverse cardiac events. Global strain reduction was also detected in patients with cancer who developed myocarditis after receiving immunotherapy with immune checkpoint inhibitors^44^.

There were limitations as follows. This was a cross-sectional single-center study. To comprehensively compare and investigate the subtle change of LV myocardium due to COVID-19 vaccination, a prospective study should be performed by planning to record prospectively an echocardiographic examination just before vaccination and then within 6 weeks afterward in the follow-up, to construct an adequate prospective comparison between symptomatic and control groups. Moreover, a larger number of study participants should be investigated further to strengthen the power of the results. In this regard, the correlation between vaccine types and dosage was not defined. The number of each vaccine type and the various combinations of mixed vaccinations in each individual was complicated to statistically quantify. The number of medications used in the study population was low because our study design aimed to exclude confounding factors such as significant cardiovascular diseases to evaluate the real cardiac AEs from vaccination. Thus, the majority of individuals had no history of medication use during the study period. In our study, the population recruitment strategy targeted two varied groups. We acknowledge the potential for selection bias. Even though all participants were recruited from the same hospital, the symptomatic group came from the cardiology clinic while the control group came from the health check center. To comprehensively identify the cardiac adverse effects of vaccination, it is necessary to consider an ideal control group of individuals who have never received the COVID-19 vaccine. However, due to Taiwan's government policy mandating vaccination for the general public, adherence to this regulation was widespread. This methodological choice was primarily driven by the need to maintain consistency in clinical evaluation and echocardiographic analysis, ensuring that all participants were subjected to the same standard of care and assessment protocols. This bias may manifest due to the differential health-seeking behaviors between the asymptomatic individuals attending routine check-ups and symptomatic patients seeking clinical care, potentially impacting the study's generalizability. However, this recruitment strategy was essential for capturing the nuanced spectrum of cardiac responses to COVID-19 vaccination, particularly given the novel nature of this investigation. By recruiting from these distinct populations, we aimed to enhance the study's relevance to clinical practice, enabling a broader understanding of vaccine-related cardiac outcomes. Furthermore, the categorization of participants into symptomatic and control groups was underpinned by rigorous clinical assessments, ensuring a robust delineation based on objective echocardiographic evidence of myocardial strain variations.

Our study observed a relatively large SD in the mean values of both GLS and GCS, accompanied by an overlap in SD between the compared groups. This variability, particularly in the context of speckle tracking echocardiography, may be influenced by several factors intrinsic to the methodology, including image quality, loading conditions, and the echocardiography system used. The analysis of LV global strains could partially stem from the heterogeneity in chest wall conformation among our study participants. Given the influence of chest wall conformation on echocardiographic reproducibility, it is imperative to consider the potential impact of chest wall conformation on the measurements. Future studies may benefit from incorporating the ratio of chest transverse diameter over the distance between sternum and spine, Modified Haller Index, as a covariate in their analyses or selecting echocardiographic methods that are less susceptible to the influence of chest wall conformation. This approach might enhance the precision of assessing myocardial strain and, by extension, the accuracy of detecting subtle cardiac changes post-vaccination^45^. However, our results of strain analysis demonstrated their properties as myocardial mechanical indicators and may give a general suggestion at the population level including awareness of transient cardiac discomforts after COVID-19 vaccination. Notably, the methodology of data collection for incidents of cardiac complications after COVID-19 vaccination independently relied on verbal questionnaires, which are relatively subjective. Even though some patients in the symptomatic group exhibited arrhythmias and sinus tachycardia, there was no evidence indicating the involvement of cardiac inflammatory markers. The correlation of strain analysis and cardiovascular magnetic resonance, the standard imaging modality, should be validated. Last, there are several available systems for strain analysis. 2D-STE implementation with different machine vendors/software would be better performed to compare their results and to reduce technical variability.

## Conclusions

The WHO stated that the development of vaccines has been shown to be the only effective tool to combat the situation. The benefits of vaccines outweigh the potential risk of vaccine-related adverse events. This study investigated cardiac manifestations, which may occur after COVID-19 vaccination evaluated by 2D-STE at first. We demonstrated that tissue speckle tracking performed well as a non-invasive, cost-effective, and valuable tool for myocardial strain analysis. Our findings suggested that GLS and GCS may be advantageous parameters utilized to evaluate the changes of the heart in patients with cardiac discomfort from COVID-19 vaccination.

## Data Availability

The datasets used and/or analyzed during the current study are available from the corresponding author upon reasonable request.
